# A commentary on ‘Circulating 25-hydroxyvitamin D concentration can predict bowel resection risk among individuals with inflammatory bowel disease in a longitudinal cohort with 13 years of follow-up’

**DOI:** 10.1097/JS9.0000000000001506

**Published:** 2024-05-03

**Authors:** Haoyu An, Hao Chi, Jie Liu

**Affiliations:** a School of Medicine, The Chinese University of Hong Kong, Shatin, New Territories, Hong Kong; b Clinical Medical College, Southwest Medical University, Luzhou; c Department of General Surgery, Dazhou Central Hospital, Dazhou, People’s Republic of China

*Dear Editor,*

A recent study by Dan *et al*.^1^ published in the *International Journal of Surgery* was read with keen interest. This research, which delved into the association between serum level of 25-hydroxy vitamin D and bowel resection risk in inflammatory bowel disease (IBD) using the data from the UK Biobank, stands out for its comprehensive statistical approach and the valuable insights provided. The primary finding highlights that low serum 25-hydroxy vitamin D concentration, especially under 50 nmol/l, is a strong independent risk factor for bowel resection risk in IBD. While the rigorous efforts and important contributions of this study are deeply appreciated, some constructive suggestions are offered for further investigation.

Firstly, the choice of concentration range in the primary analysis is considered. It is acceptable to divide the serum 25-hydroxy vitamin D concentration into 10–29.3, 29.3–41.4, 41.4–53.1, 53.1–66.5, and 66.5–148 nmol/l, while it would be beneficial if the rationale behind these specific ranges were elucidated in greater detail. An alternative range might potentially serve as a better predictive factor.

Secondly, it is missing some important confounding factors. In the multivariate analysis, the study has commendably constructed three models to perform stepwise regression. However, the air pollution factors reported to potentially influence the risk of IBD-related surgery^2^, also mentioned in the introduction part, were neither assessed in the assessment of covariates nor considered in the regression models. Air pollution data including nitrogen dioxide, particulate matter 2.5 μm, as well as particulate matter 10 μm are accessible through the UK Biobank.

Lastly, the analysis yielded an intriguing result. When comparing extreme quintiles of 25(OH)D level, participants with IBD showed a 34% reduced risk of bowel resection (95% CI: 11%–51%, *P*=0.007), while this association was not significant in Crohn’s disease (HR=0.93, 95% CI: 0.59–1.45, *P*=0.740). The reasons for this phenomenon should be further investigated and discussed. Perhaps the best subgroup is not in extreme quintiles.

In summary, the study by Dan *et al*. is a significant step forward in understanding the risk factors for bowel resection risk in IBD. Such investigations can pave the way for future preventive and therapeutic strategies and better predictive models, potentially benefiting a vast number of individuals. Our suggestions are merely to refine an already outstanding piece of research further. We eagerly await more excellent works from the authors in the future.

## Ethical approval

Not applicable.

## Consent

Not applicable.

## Sources of funding

The study was approved by the Dazhou Science and Technology Bureau project (21ZDYF0025, 21ZDYF0023), the Sichuan Provincial Administration of Traditional Chinese Medicine project (2023MS141), and the Sichuan Medical Association project (S21048).

## Author contribution

J.L.: conceptualization; H.A. and H.C.: formal investigation, and writing; J.L.: supervision. All authors commented on previous versions of the manuscript. All authors read and approved the final manuscript.

## Conflicts of interest disclosure

The authors declare no conflicts of interest.

## Research registration unique identifying number (UIN)

Not applicable.

## Guarantor

Jie Liu.

## Data availability statement

Data sharing is not applicable to this correspondence as no datasets were generated or analyzed.

## Provenance and peer review

Not applicable.
